# Acidic Microenvironment Determines Antibiotic Susceptibility and Biofilm Formation of *Pseudomonas aeruginosa*

**DOI:** 10.3389/fmicb.2021.747834

**Published:** 2021-11-19

**Authors:** Qiao Lin, Joseph M. Pilewski, Y. Peter Di

**Affiliations:** ^1^Department of Environmental and Occupational Health, University of Pittsburgh, Pittsburgh, PA, United States; ^2^Division of Pulmonary, Allergy, and Critical Care Medicine, Department of Medicine, University of Pittsburgh, Pittsburgh, PA, United States

**Keywords:** cystic fibrosis - CF, *Pseudomonas aeruginosa*, antibiotic resistance, bacterial evolution, acidic pH

## Abstract

*Pseudomonas aeruginosa* is the most prevalent bacterial species that contribute to cystic fibrosis (CF) respiratory failure. The impaired function of CF transmembrane conductance regulator leads to abnormal epithelial Cl^–/^HCO_3_^–^ transport and acidification of airway surface liquid. However, it remains unclear why the CF lung is most commonly infected by *Pseudomonas aeruginosa* versus other pathogens. We carried out studies to investigate if lower pH helps *Pseudomonas aeruginosa* adapt and thrive in the CF-like acidic lung environment. Our results revealed that *Pseudomonas aeruginosa* generally forms more biofilm, induces antibiotic resistance faster in acidic conditions, and can be reversed by returning the acidic environment to physiologically neutral conditions. *Pseudomonas aeruginosa* appears to be highly adaptive to the CF-like acidic pH environment. By studying the effects of an acidic environment on bacterial response, we may provide a new therapeutic option in preventing chronic *Pseudomonas aeruginosa* infection and colonization.

## Introduction

Cystic fibrosis (CF) is a genetic disease involving compromised function of cystic fibrosis transmembrane conductance regulator (CFTR) that leads to impaired airway host defense and therefore causes lung inflammation and bacterial infections (Pezzulo et al., 2012; Shah et al., 2016). The environment in CF lungs is thought to be acidic, as evidenced by the lower-than-neutral pH value of the airway surface liquid (ASL) in newborn CF pigs and differentiated human and porcine primary epithelial cell cultures, compared to non-CF controls (Pezzulo et al., 2012; Shah et al., 2016). The malfunction of CFTR also leads to elevated Na^+^ and Cl^–^ levels in the airway, inhibiting the natural antimicrobial factors in ASL (Zabner et al., 1998). *Pseudomonas aeruginosa* (*P. aeruginosa*) is an opportunistic Gram-negative pathogen that is the most prevalent bacterial species in adult CF lungs and contributes to the associated high mortality rates (Silva Filho et al., 2013). The prevalence of *P. aeruginosa* lung infection gradually increases over time from approximately 20% to 70% in CF patients from age 2 to 45 (Cystic Fibrosis Foundation, 2020), which coincides with an increase in CF disease severity. Bacteria expand their population in the natural environment and the host via two different forms. The planktonic form of free-moving bacteria is the typical way bacteria spread themselves at the initial stage of reaching a new environment. In contrast, the accumulated form of bacteria, so-called “biofilm,” represents another critical pathogenic mechanism. It is noted that the bacterial biofilm formation in the CF airway likely contributes to the chronic colonization of *P. aeruginosa* (Lyczak et al., 2002).

Many factors contribute to CF airway acidification, and there are adverse health consequences. From the bacterial perspective, it has been reported that extracellular DNA (eDNA) is the most abundant polymer within the *P. aeruginosa* biofilm matrix (Matsukawa and Greenberg, 2004; Okshevsky and Meyer, 2015). Previous studies suggested that eDNA acidifies *P. aeruginosa* biofilm and promotes resistance to aminoglycoside antibiotics (Wilton et al., 2016). Studies also demonstrated that when *P. aeruginosa* is under acidic stress, the bacterial outer membrane permeability (PhoPQ/PmrAB-controlled surface modifications) could decrease and result in decreased antibiotic uptake (Adewoye and Worobec, 1999; Wilton et al., 2016). Additionally, acidic pH likely modulates aminoarabinose-modified LPS and spermidine, which mask bacterial negative surface charges and block the entrance of aminoglycosides (Ernst et al., 2007; Johnson et al., 2012; Wilton et al., 2016). Our results demonstrated that *P. aeruginosa* grew under other classes of antibiotics (β-lactam and fluoroquinolone), in addition to the previously published aminoglycosides (Wilton et al., 2016), also increase the acquired antibiotic tolerance. Currently, known mechanisms could partly explain why acidic pH induces immediate antibiotic tolerance, but the long-term impact of acidic pH on bacterial evolution with antibiotic treatment remained unknown. From the host perspective, the CF lung is burdened with acidic eDNA originated from the immune cells recruited by chronic bacterial infections (Lethem et al., 1990). In addition, Shah et al. (2016) reported evidence that the non-gastric H+/K+ adenosine triphosphatase (ATP12A), a proton pump that actively acidifies human and porcine airways, is absent in mice. This explains why CFTR-deficient mice with no acidic airway epithelium are free from opportunistic respiratory infections.

The evidence of CF airway acidification and the high prevalence of *P. aeruginosa* among CF lung infections prompted us to determine the effects of a CF-like acidic environment on the pathogenic ability of *P. aeruginosa*. Our results indicated that the acidic environment stimulates increased *P. aeruginosa* biofilm formation, promotes faster bacterial evolution toward elevated antibiotic resistance as evidenced by stable genetic mutations, and increases expressions of multiple biofilm/virulence-related genes. Interestingly, based on our observation, *P. aeruginosa* was the only bacterial species from the notorious ESKAPE pathogens (*Enterococcus faecium, Staphylococcus aureus, Klebsiella pneumoniae, Acinetobacter baumannii, Pseudomonas aeruginosa*, and *Enterobacter* spp.) that showed a pattern of increased biofilm formation in acidic conditions. Furthermore, the acidic environment on the apical surface of differentiated primary bronchial epithelial cells isolated from CF patients (CFBEs) results in increased *P. aeruginosa* attachment and bacterial numbers than the physiologically neutral environment on the surface of cultured primary human bronchial epithelial cells from normal subjects (HBEs). These adverse effects of a CF-like acidic environment can be ameliorated by modulating the acidic environment into physiologically neutral conditions. The varying characteristics and behavior of *P. aeruginosa* in different pH conditions may provide additional treatment targets and options for CF sufferers in preventing chronic *P. aeruginosa* infection and colonization.

## Results

### The Response of *Pseudomonas aeruginosa* to Cystic Fibrosis-Like Microenvironment

*Pseudomonas aeruginosa* gradually increases its presence in the CF airways after the pathological phenotypes of CFTR malfunction appear more obviously with the increased age of the CF sufferers (Cystic Fibrosis Foundation, 2020). It is well-documented that compromised functions of CFTR and the CFTR-modulated HCO_3_^–^ secretion in CF result in an abnormal environment of higher salt (∼100 ± 5 mM) (Zabner et al., 1998) and lower pH (∼6.7 ± 0.3) (Chen et al., 2010; Pezzulo et al., 2012; Shah et al., 2016) in the CF ASL than those of normal subjects.

We first examined the effects of salt concentration and acidic condition on bacterial planktonic and biofilm mode of growth to understand the effects of the CF-like microenvironment on the behavior of *P. aeruginosa*. Two widely used *P. aeruginosa* lab strains (PAO1 and PA14) and two multi-drug resistant (MDR) *P. aeruginosa* clinical strains isolated from CF patients (*P.a.* 129-5 and *P.a.* 152-19) were selected for this experiment. The growth curves indicated that various salt concentrations (addition of 50, 100, and 150 mM NaCl) and pH conditions (pH = 6.0, 6.5, 7.0, and 7.5 adjusted by HCl) did not result in any noticeable difference in the proliferation rate of planktonic *P. aeruginosa* in all four strains (Supplementary Figure S1). There was less biofilm biomass formed with the increasing NaCl concentrations in PAO1. In contrast, the effect of salt concentrations on biofilm formation was not apparent in the other three *P. aeruginosa* strains (Supplementary Figure S2). Nonetheless, we observed significant changes in the initial attachment of bacterial biofilm under acidic pH conditions after just 3 h of incubation (Supplementary Figure S2). Except for PA14, all the other three tested *P. aeruginosa* strains showed similar results of increased attachment of bacteria at acidic (pH < 7) conditions within each tested NaCl concentration group. These results suggest that the acidic environment likely promoted the initial attachment of *P. aeruginosa*.

### Increased *Pseudomonas aeruginosa* Antibiotic Tolerance Under Acidic pH Conditions

**FIGURE 1:**
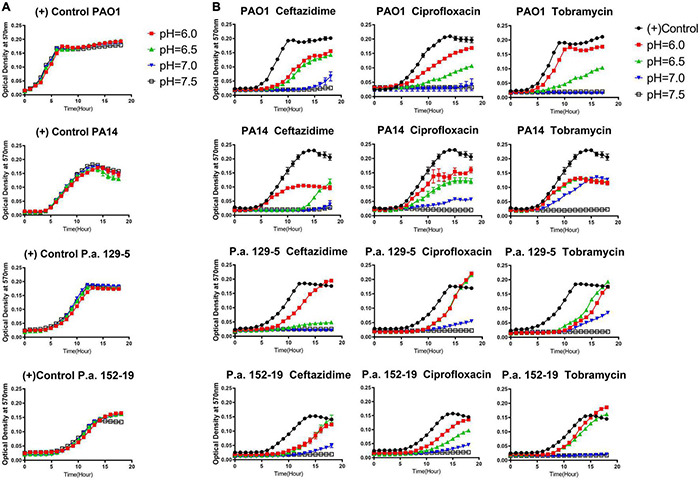
Acidic pH conditions impair the antimicrobial activity of standard-of-care antibiotics against *P. aeruginosa*. **(A)** *P. aeruginosa* growth curve in pH adjusted, antibiotic-free medium. **(B)** *P. aeruginosa* growth curve in pH adjusted medium, supplemented with ceftazidime, ciprofloxacin, and tobramycin at the respective MIC for each antibiotic at pH 7.5. A pH-adjusted 10% TSB medium was used in this experiment. Optical density at 570 nm was measured every hour for 18 h at 37°C in a microplate reader. A closely fitted lid was placed on the microplate to prevent liquid evaporation. No samples were placed near the edge of the 96-well microplate to prevent any drying effect during overnight incubation. Results are mean ± SEM from three independent experiments with bacteria grew in duplicates for each condition.

It is known that common antibiotic treatments such as inhaled tobramycin and ceftazidime in CF respiratory infections do not guarantee respiratory *P. aeruginosa* eradication (Mayer-Hamblett et al., 2012; Cystic Fibrosis Foundation, 2020). Thus, we sought to determine if the CF-like acidic environment enhanced bacterial tolerance to antibiotics. Three different classes of commonly used standard-of-care antibiotics, including ceftazidime (β-lactam), ciprofloxacin (fluoroquinolone), and tobramycin (aminoglycosides), each with a distinct antibacterial mechanism, were tested against the same four bacterial strains of *P. aeruginosa* at their respective MICs (at physiologically neutral pH 7.5) under different pH conditions. Our results indicated that the acidic pH environment alone does not notably change the *P. aeruginosa* proliferation rate (Figure 1A). However, acidic conditions increased bacterial tolerance to all three clinically used antibiotics, as demonstrated in the elevated growth curves compared to pH 7.5 when the same dosage of each antibiotic was used in all varying pH conditions (Figure 1B). In all cases, *P. aeruginosa* grown under the acidic conditions (pH = 6.0 and pH = 6.5) demonstrated higher tolerance to the antibiotic treatments as their growth curves were closer to those of the “no antibiotic treatment” controls. The slope of the growth curve gradually decreases as the pH value increases. To determine if the increased bacterial growth in lower pH conditions was due to permanent degradation or temporary inactivation of antibiotics in the acidic environment, we pre-incubated the antibiotics in physiologically neutral pH 7.5 or acidic pH 6.0 for 5 h before the bacterial growth kinetic experiments. Interestingly, the antibiotics pre-exposed to pH 6.0 and pH 7.5 showed similar growth inhibition curves when the growth inhibition assay (GIA) was performed at the pH 7.5 condition (Supplementary Figure S3). The results indicated that antibiotics pre-exposed to acidic conditions (pH = 6.0) did not lose their drug potency and regained their antibacterial activity after returning to the physiologically neutral condition (pH = 7.5). These data suggested that *P. aeruginosa* could quickly become more tolerant to antibiotic treatment in an acidic environment.

### Increased *Pseudomonas aeruginosa* Antibiotic Resistance Under Acidic pH Conditions

**FIGURE 2:**
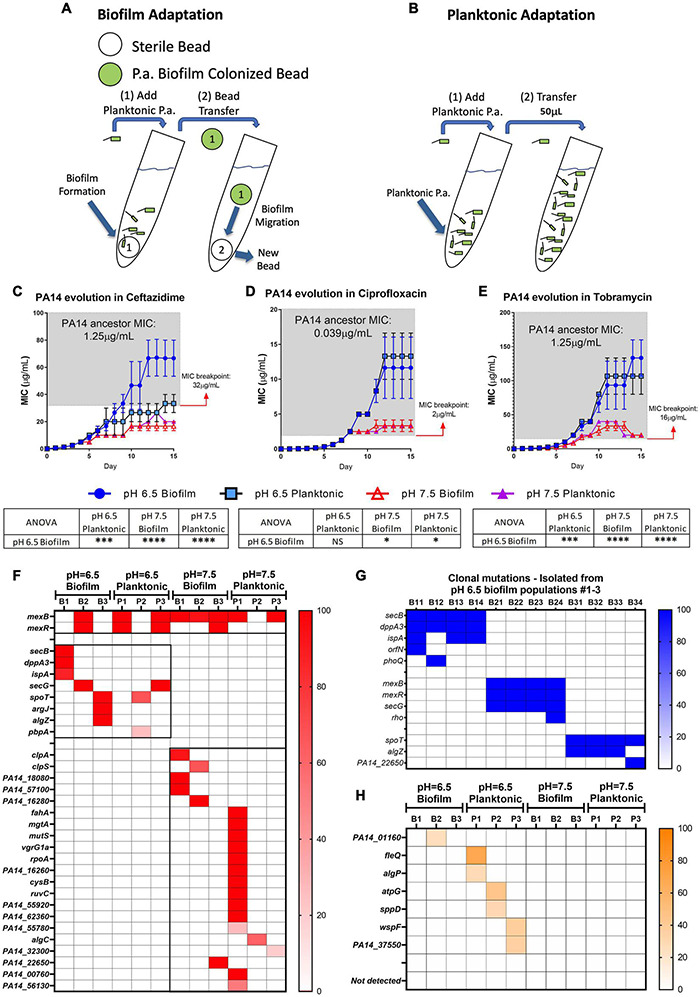
Acidic pH promotes faster accumulation of adaptive resistance of *P. aeruginosa* against antibiotics compared to pH 7.5. **(A,B)** Schematics of the *P. aeruginosa* PA14 antibiotic adaptation experiment: biofilm on beads **(A)** and planktonic bacteria **(B)** were transferred to fresh m63 media (pH = 6.5/7.5, adjusted by HCl) every 24 h. **(C–E)** The PA14 biofilm/planktonic cultures were treated with 1/2 MIC at day 1. Antibiotic concentrations were doubled after every bead/planktonic transfer (*n* = 3). The gray boxes on panels **(C–E)** denote MIC values that are considered antibiotic-resistant according to the guideline “MIC Breakpoints for *Pseudomonas aeruginosa*,” published by The Clinical and Laboratory Standards Institute. **(F)** PA14 population mutations were identified after 15 days of evolution with ceftazidime. Each bacterial lifestyle/pH was evolved in triplicate populations. Biofilm (B)/planktonic (P) populations were labeled as B1, B2, B3, and P1, P2, P3, respectively. **(G)** Clonal mutations detected after 15 days of evolution with ceftazidime. Clones were labeled from number 1 to 4 following their population number. For example, B11 represents biofilm population B1, clone #1. **(H)** Mutations from PA14 evolution without antibiotics for 15 days. Color scale bars represent mutation frequency, which ranges from 0 to 100%. Data are mean ± SEM. One-way ANOVA was performed by comparing the combined day 14 and 15 MIC values. ^∗^*p* < 0.05; ^∗∗∗^*p* < 0.001; ^∗∗∗∗^*p* < 0.0001; NS, not significant.

To further explore if acidic pH promotes *P. aeruginosa* antibiotic resistance, we carried out studies to investigate bacterial evolution with antibiotics in acidic and neutral pH conditions. Both biofilm and planktonic *P. aeruginosa* were grown under the treatment of ceftazidime, ciprofloxacin, and tobramycin, three of the most commonly used antibiotics in treating CF respiratory infection. The reference strain of *P. aeruginosa* (PA14) was selected because we sought to focus on investigating the pH effect without concerns of horizontally transferred plasmids or bacteriophages that could exist in clinical strains. The *P. aeruginosa* biofilm was generated by growing PA14 on acrylic beads and transferred daily to a new culture tube with a new sterile bead in the existence of antibiotics (Figure 2A). The planktonic culture served as a control of the biofilm evolution model in contrasting the different growth modes (Figure 2B). The new MIC of each PA14 culture condition was determined daily by the survival of each population after 24 h of incubation in a fresh medium containing doubled antibiotic concentrations. After 15 days of continuous growth and evolution, PA14 populations acquired increased levels of resistance against all three antibiotics. The acidic environment significantly increased the MIC required for the tested antibiotics to kill *P. aeruginosa* (Figures 2C–E), regardless of the antibiotic killing mechanisms.

Acidic pH environment induces immediate antibiotic tolerance of *P. aeruginosa* in planktonic growing conditions up to eightfold compared to a neutral pH environment (Supplementary Table S1). Furthermore, we observed a 32- to 512-fold MIC increase in the acidic pH treated biofilm populations after 15 days of evolution (Figures 2C–E). Therefore, we hypothesized that PA14 underwent genetic mutations to survive antibiotic pressure, and the ceftazidime treated PA14 populations were selected for further analysis. Whole-genome sequencing (WGS) of the bacteria was performed in every testing bacterial population to identify the high-frequency mutations (Figure 2F). WGS of four randomly picked single colonies from each of the pH 6.5 biofilm populations (B1–B3) were then carried out to directly determine the evolved genotypes in correspondence to the MIC-increasing phenotypes (Table 1). Common mutations on the efflux pump (*mexB* and *mexR*) known to induce ceftazidime resistance (Sanz-Garcia et al., 2018) were identified in half of pH 6.5 populations and five out of six pH 7.5 populations (Figure 2F). The *mexB* and *mexR* mutations conferred pH 6.5 population B2 ceftazidime resistance a 64-fold increase (Figure 2C), which was subsequently confirmed by clonal genotype (Figure 2G) and MIC (Table 1) of selected individual clones.

**TABLE 1:** PA14 clonal MIC from the ceftazidime evolution study^∗^.

**Population**	**Clone number**	**Clonal MIC (fold increase)**		**pH-induced fold increase**
		**pH 6.5**	**pH 7.5**	
Biofilm pH 6.5 population 1 (B1)	B11	64	4	16
	B12	80	4	20
	B13	64	4	16
	B14	94	6	15.7

Biofilm pH 6.5 population 2 (B2)	B21	96	10	9.6
	B22	96	24	4
	B23	96	24	4
	B24	128	32	4

Biofilm pH 6.5 population 3 (B3)	B31	6	4	1.5
	B32	4	4	1
	B33	6	4	1.5
	B34	8	4	2

*^∗^Ceftazidime MIC against PA14 ancestor: 1.25 μg/mL.*

Interestingly, three out of six of the evolved bacterial populations under the pH 6.5 conditions (biofilm and planktonic) generated antibiotic resistance to ceftazidime via efflux pump-independent mechanisms. For instance, the combined mutations in *secB* and *dppA3* resulted in an acidic pH-inducible 64-fold MIC increase in the pH 6.5 biofilm population B1 (Figure 2C) and a 64- to 94-fold MIC increase in individual clones (Figure 2G and Table 1). Mutations on *spoT, argJ*, and *algZ* potentially contributed to a 32-fold MIC increase in the pH 6.5 biofilm population B3 but in the selected individual clones only showed mild MIC increase (4- to 8-fold increase, Table 1), which indicated a common discrepancy in MIC due to bacterial lifestyle difference. The sequencing results indicated that the mutations generated from PA14 bacterial populations evolved without antibiotics (Figure 2H) do not overlap with mutations identified under antibiotic selection (Figures 2F,G), which suggests that acidic pH condition is a critical factor in promoting the evolution of drug-resistance when antibiotics are present.

### Acidic Conditions Promote Biofilm Formation of *Pseudomonas aeruginosa* Clinical Isolates and Compromise Biofilm Prevention Activities of Antibiotics

**FIGURE 3:**
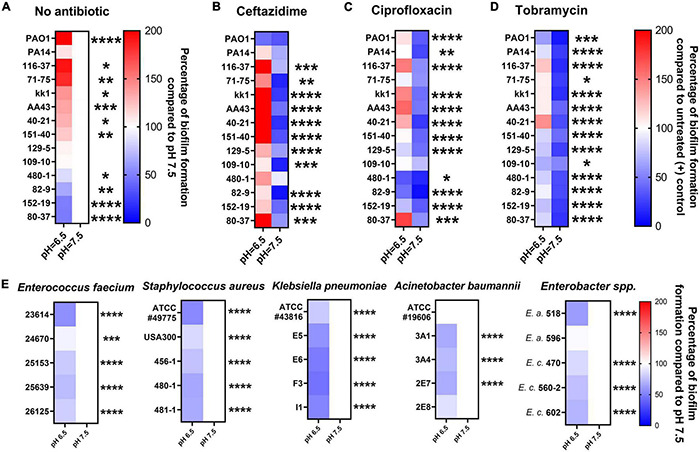
Acidic pH modulates ESKAPE pathogens biofilm formation and impairs antibiotic biofilm prevention against *P. aeruginosa.* All *P. aeruginosa* strains (2 lab strains and 12 clinical strains) were incubated in pH adjusted m63 medium for 18 h. The crystal violet staining method was used to quantify biofilm formation (*n* = 6). **(A)** *P. aeruginosa* biofilm formation without antibiotic treatment. Biofilm formation in pH 7.5 served as a positive control. **(B–D)** *P. aeruginosa* biofilm formation was treated by ceftazidime, ciprofloxacin, and tobramycin at the concentrations of 1x MIC of each *P. aeruginosa* strain (MIC in normal pH 7.5 condition). The percentage of antibiotic-treated biofilm formation was calculated by comparing it to each untreated *P. aeruginosa* strain in pH 7.5. **(E)** Biofilm formation of other bacterial species of the ESKAPE bacterial pathogens. Five isolates from each of the other ESKAPE species were grown at pH 6.5 and 7.5 (as a control) in biofilm mode. *E.a.* and *E.c.* are abbreviations for *Enterobacter aerogenes* and *Enterobacter cloacae*, respectively. The color scale bars represent the percentage of biofilm formation compared to appropriate control groups. Red scale: increased biofilm formation (100%–200%); white: no change (100%); blue scale: decreased biofilm formation (0–100%). Data were collected from three independent experiments. Unpaired *t*-test was used for statistical analysis between each pH 6.5 and pH 7.5 conditions. ^∗^*p* < 0.05; ^∗∗^*p* < 0.01; ^∗∗∗^*p* < 0.001; ^∗∗∗∗^*p* < 0.0001; otherwise not significant.

We further evaluated fourteen *P. aeruginosa* strains to validate the hypothesis that acidic pH promotes bacterial biofilm formation and impairs antibiotic efficacy. These include twelve clinical *P. aeruginosa* isolates obtained initially from CF patients and two standard lab strains (PAO1 and PA14). In Figure 3A, the CF-like, acidic pH 6.5 condition alone stimulated more mature biofilm formation from approximately 71% (10/14) of all *P. aeruginosa* strains compared to pH 7.5. The difference is significant and more noticeable when 1x MIC of ceftazidime, ciprofloxacin, and tobramycin was used in *P. aeruginosa* biofilm prevention. In Figures 3B–D, 14 *P. aeruginosa* strains were treated with antibiotics using their respective MIC dosages (Supplementary Table S1) in pH 6.5 and 7.5 conditions. The majority of antibiotic-treated *P. aeruginosa* strains formed more biofilm after 18 h in the pH 6.5 condition compared to the pH 7.5 condition. These biofilm data are in accordance with our growth kinetic studies and the PA14 evolution studies (Figures 1, 2). All experiment results showed a similar pattern that ceftazidime, ciprofloxacin, and tobramycin were generally less effective in inhibiting *P. aeruginosa* growth and preventing biofilm formation at acidic pH conditions. All other five species of ESKAPE pathogens except *P. aeruginosa* conversely demonstrated decreased biofilm formation under acidic pH stress (Figure 3E).

### Neutralization of Acidic pH in Cystic Fibrosis Epithelial Cells Restores Impaired Host Defense

**FIGURE 4:**
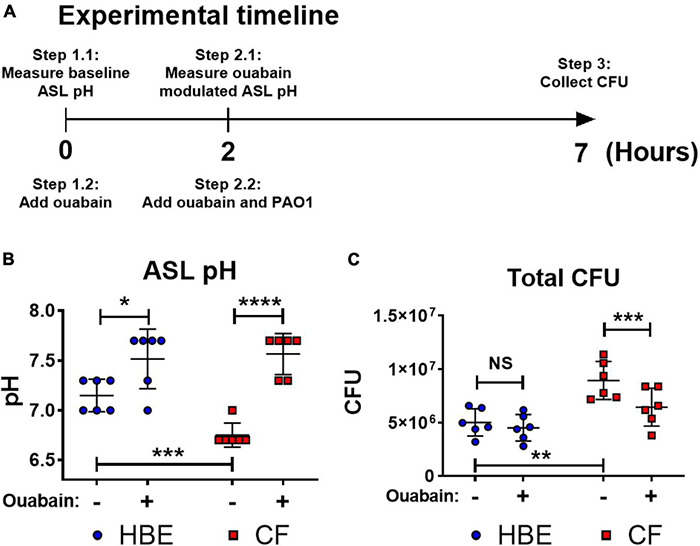
Ouabain helps restore host defense activities by reversing the acidic pH to neutral pH in differentiated human bronchial epithelial cell cultures. **(A)** Experimental timeline. pH values were measured on the apical side of air-liquid interface cultured cells before and after 2 h of treatment with/without 20 μM ouabain; PAO1 was then added to the apical side of cultured cells with ouabain or DMSO solvent control for additional 5 h. **(B)** Effect of ouabain treatment on pH values in non-CF (HBE) and CF epithelial cells. The change of pH in the epithelial apical wash was measured by narrow range pH test strips (pH 6–7.7, resolution 0.3 pH unit; *n* = 6). **(C)** Effect of ouabain treatment on *P. aeruginosa* CFU in non-CF (HBE) and CF epithelial cells. PAO1 was incubated on the apical side of cultured HBE and CF epithelial cells in the existence of 20 μM ouabain or DMSO (solvent control). Biofilm and planktonic CFU were obtained by plated on agar plates for total PAO1 CFU (*n* = 6). Data were collected from two independent experiments. Unpaired *t*-test was used for statistical analysis. Data are mean ± SEM. ^∗^*p* < 0.05; ^∗∗^*p* < 0.01; ^∗∗∗^*p* < 0.001; ^∗∗∗∗^*p* < 0.0001; NS, not significant.

Acidic pH impairs important host defense mechanisms such as the ASL antibacterial activities of antimicrobial peptides, including β-defensin-1, -3, and LL-37 (Johansson et al., 1998; Nakayama et al., 2002; Abou Alaiwa et al., 2014). Our results demonstrate that the acidic pH in the CF-like microenvironment increases bacterial tolerance/resistance against antibiotics and enhances biofilm formation, which may promote *P. aeruginosa* colonization. To simulate the airway microenvironment, we further determined if the neutralization of the acidic ASL of differentiated human bronchial epithelial cells that were maintained under air–liquid interface (ALI) culture could help alleviate *P. aeruginosa* infection (Figure 4A). The primary human bronchial epithelial (HBE, non-CF) cells and CF bronchial epithelial (CFBE) cells were used. We tested ouabain, an ATP12A inhibitor that inhibits H^+^ secretion by human epithelial cells, raising the pH value of cultured epithelial cells (Shah et al., 2016). Without any treatment, the ASL of cultured CFBE cells was more acidic than non-CF HBE cells (Figure 4B). However, ouabain successfully elevated the pH value of CFBE ASL from approximately 6.7 to 7.6. The increased pH value reflected neutralized ASL of CFBE, which consequently resulted in decreased CFU of *P. aeruginosa* (PAO1) grew on top of human epithelial cells (Figure 4C). The ouabain modulation of pH in ASL of HBE was minimal, which did not result in significant changes of *P. aeruginosa* CFU (Figure 4C). Ouabain itself does not display any bactericidal activity at the treatment concentration of 20 μM (Supplementary Figure S4).

### Bacterial Gene Expressions Are Modulated by Acidic pH

**FIGURE 5:**
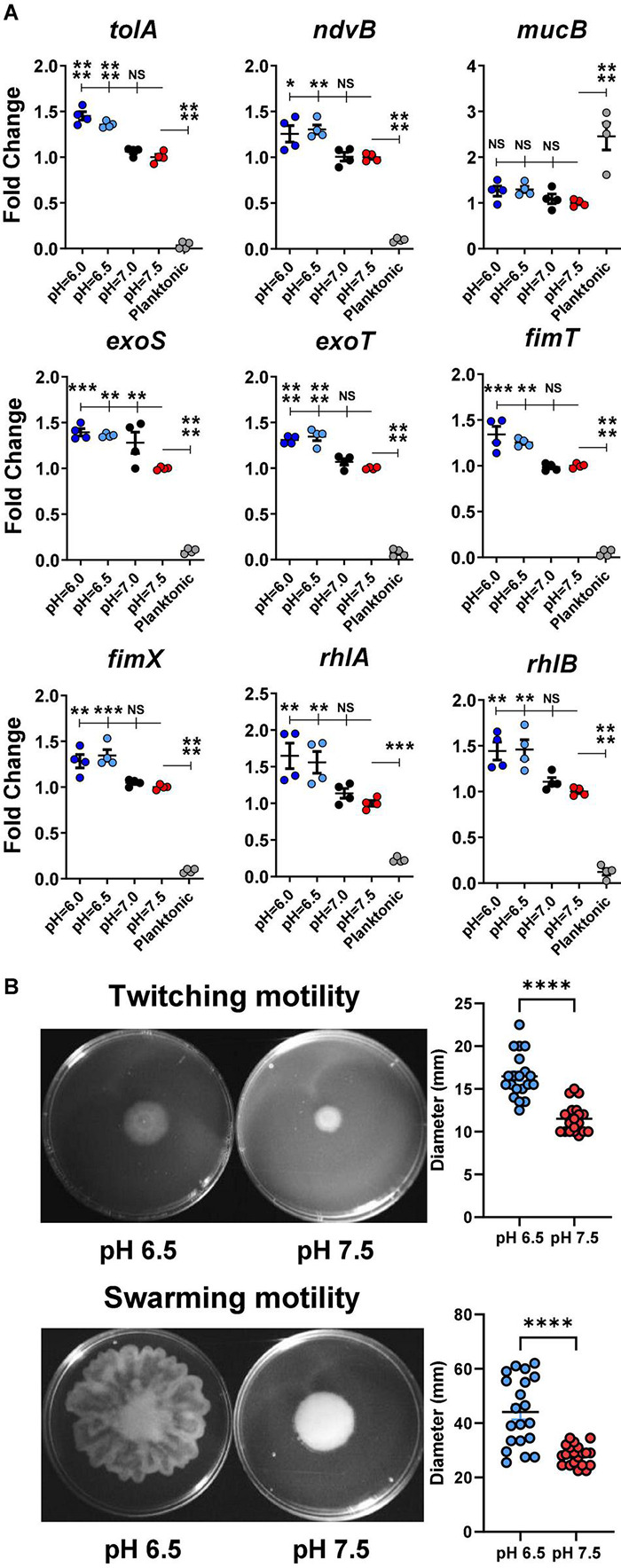
Acidic pH conditions increase biofilm/virulence-related gene expression of *P. aeruginosa*. **(A)** *P. aeruginosa* PAO1 biofilm was cultured in pH adjusted DMEM for 3 h. Physiologically related biofilm condition in pH 7.5 was used as a control for biofilm growth. Planktonic form of *P. aeruginosa* inoculated in pH 7.5 was also included for comparison (*n* = 4). **(B)** PAO1 motility assays. PAO1 swarming and twitching motility assays were performed on pH adjusted M8 agar (*n* = 20). Data were representative of two independent experiments. Results are mean ± SEM. One-way ANOVA and Dunnett’s multiple comparisons test were used for gene expression statistical analysis. Student’s *t*-tests were used for bacterial motility assay statistical analysis ^∗^*p* < 0.05; ^∗∗^*p* < 0.01; ^∗∗∗^*p* < 0.001; ^∗∗∗∗^*p* < 0.0001; NS, not significant.

To evaluate if acidic microenvironment-enhanced *P. aeruginosa* infection in CF is regulated through increased bacterial biofilm formation at the transcriptomic level, we determined the gene expression of a panel of biofilm/virulence-related genes (Figure 5A) in association with the observed biomass changes. Multiple biofilm-related genes such as *tolA, ndvB, rhlA*, and *rhlB* were all significantly increased in the acidic conditions of biofilm formation.

Additionally, the expressions of several virulence-associated genes such as *exoS, exoT, fimT*, and *fimX* were also significantly increased in acidic environments (pH 6.0 and 6.5) compared to in physiological conditions (pH 7.0 and 7.5). Interestingly, the planktonic-associated mucB gene expression did not vary among all acidic and physiological biofilm-forming pH conditions. However, its expression differed significantly between the biofilm and planktonic forms of *P. aeruginosa*.

The *rhlA* and *rhlB* controlled rhamnolipid biosurfactant synthesis (Caiazza et al., 2005), and their gene expression data were validated by a bacterial swarming motility assay (Figure 5B). *P. aeruginosa* (PAO1) displayed significantly increased swarming diameter on acidic agar compared to pH 7.5 agar. Similarly, the results from the follow-up twitching motility assay agreed with the elevated expression *fimT* and *fimX* in acidic pH conditions (Figure 5B).

## Discussion

*Pseudomonas aeruginosa* is the most dominant bacterial pathogen associated with CF disease severity and mortality. The CF microenvironment resulted from a CFTR malfunction may have an unwanted effect in contributing to the infection and colonization of *P. aeruginosa*. However, the underlying pathogenic mechanisms remain to be elucidated. In this study, we investigated the effect of CF-like acidic microenvironment on the behavior of *P. aeruginosa*. We demonstrated that multiple *P. aeruginosa* clinical isolates significantly increased their biofilm formation and antibiotic tolerance/resistance under acidic conditions. Remarkably, this pathological factor of acidic pH also activated a series of biofilm- and virulence-related genes of *P. aeruginosa*.

Under the selection pressure in an experimental condition with gradually increased antibiotic concentrations, we demonstrated that *P. aeruginosa* adapted to the antibiotics quickly and evolved significantly faster in pH 6.5 than in pH 7.5, which eventually acquired strong antibiotic resistance in only 15 days [approximately 99 generations, estimated at 6.6 generations/day (Flynn et al., 2016)]. In contrast to the PA14 ancestor, evolved PA14 progeny bacteria demonstrated an indisputable ability to survive and adapt to highly stressful antibiotic treatments, while acidic pH significantly expedited this process. Of note, all of the frequently detected *mexB* mutations are single nucleotide polymorphisms (SNPs). These SNPs are not necessarily loss-of-function mutations, which would allow *P. aeruginosa* to maintain the MexAB-OprM efflux pump activities against ceftazidime. On the contrary, mutations on the *mexR* gene, a bacterial efflux pump repressor, are sometimes indels, which could lead to unsuppressed efflux pump activities. Both mechanisms likely contributed to the elevated PA14 ceftazidime resistance.

Our findings suggest that the acidic CF microenvironment likely plays a critical role in facilitating *P. aeruginosa* colonization despite the antibiotic treatment. Interestingly, *P. aeruginosa* appears to be the only bacterial species among the notorious MDR ESKAPE pathogens that displayed significantly increased biofilm formation under a CF-like acidic environment (Figure 3E), which may explain the high prevalence of *P. aeruginosa* in adult CF population when their airways are expected to be more acidic. Our data provide an important link between the worsened CFTR function-associated acidic microenvironment and enhanced *P. aeruginosa* biofilm formation supported by the evidence of *P. aeruginosa* biofilm-related phenotypic/genetic changes and multiple microenvironment acidification factors.

It has been reported that the lowered airway pH is associated with impaired host defense mechanisms (Johansson et al., 1998; Zabner et al., 1998; Adewoye and Worobec, 1999; Nakayama et al., 2002; Abou Alaiwa et al., 2014; Shah et al., 2016; Tang et al., 2016). By neutralizing the acidic environment via inhibiting ATP12A, the apical pH value increased on the differentiated human primary airway epithelial cells (CFBE and non-CF HBE). The neutralized pH not only can potentially restore the antimicrobial activity of the naturally secreted host defense factors by airway epithelial cells such as SPLUNC1 (Di et al., 2013; Walton et al., 2016) but also may prevent biofilm formation and/or pH-induced drug-tolerance/resistance of *P. aeruginosa*. These observations could provide potential therapeutic insights. For instance, there are several means to neutralize the acidic CF lung environment. By inhibiting ATP12A, the secretion of H^+^ is inhibited, and therefore pH is elevated (Figure 4B). The extracellular H^+^ could also be neutralized by commonly used substances, such as sodium bicarbonate (Wilton et al., 2016; Dobay et al., 2018) or hydroxide salts. The increased bacterial susceptibility to host factors after raising the acidic pH to neutral pH suggests an alternative approach in treating CF chronic infection induced by *P. aeruginosa*. Recent studies showed evidence that CFTR modulators, such as ivacaftor and lumacaftor–ivacaftor combination, increased the lung function of several genotypes of human CFTR mutations (Rowe et al., 2014; Caverly et al., 2015) and decreased *P. aeruginosa* lung culture positivity rates (Heltshe et al., 2015). The increased CFTR activity reduces sweat Cl^–^, which may subsequently result in elevated ASL pH (Abou Alaiwa et al., 2018). However, the CFTR genotypes that are not covered by the CFTR modulators continue to be a challenge for *P. aeruginosa* management.

In addition to the efforts to neutralize the acidic host environment, it is also vital to examine the complex response of *P. aeruginosa* to acidic pH and how this response leads to antibiotic tolerance/resistance. Our gene expression results provided mechanistic insight into pH-related pathways in pathogenic *P. aeruginosa*. Acidic conditions at pH values of 6 and 6.5 significantly increased the expression of *tolA*, activated in biofilms (Whiteley et al., 2001). Its product is responsible for aminoglycoside resistance in *P. aeruginosa* by decreasing its permeability and blocking the entrance of antibiotics (Bryan et al., 1976, 1980; Bryan and Kwan, 1983; Rivera et al., 1988; MacLeod et al., 2000). The *ndvB* gene encodes for a glucosyltransferase that is required for the synthesis of cyclic-b-(1, 3)-glucans (Bhagwat et al., 1996). Cyclic glucans can physically interact with tobramycin and therefore eliminate antibiotics before reaching their targeted site of action (Mah et al., 2003). In acidic conditions, there was a noticeably higher *ndvB* gene expression in *P. aeruginosa* biofilm compared to the pH 7.5 of physiological control. Planktonic form of PAO1 expressed significantly less *ndvB* than any biofilm group. The elevated expression of *ndvB* could be one factor contributing to the acidic pH-induced antibiotic resistance in *P. aeruginosa* (Figures 1–4). Both *rhlA* and *rhlB* are required for rhamnolipid synthesis (Deziel et al., 2003), a biosurfactant that contributes to the swarming motility of *P. aeruginosa* to promote biofilm colonization. Rhamnolipids are known to interfere with phagocytosis (McClure and Schiller, 1996) and normal tracheal ciliary function (Read et al., 1992). The expression of these virulence factors was increased under the acidic pH environment. Both *fimT* and *fimX* are responsible for the biogenesis and functioning of type IV pili and twitching mobility of *P. aeruginosa* (Huang et al., 2003) and are crucial during biofilm formation (O’Toole and Kolter, 1998; Chiang and Burrows, 2003). *P. aeruginosa* injects cytotoxins (e.g., ExoS and ExoT) into host cells by utilizing the type III secretion system (Barbieri and Sun, 2004). Acidic pH conditions activated *exoS* and *exoT* will likely lead to delayed wound healing, impaired phagocytosis, and spread of *P. aeruginosa* (Hauser, 2009). The *mucB* gene is a negative regulator of the sigma factor AlgU. Inactivation of this gene in acidic conditions will facilitate the conversion of *P. aeruginosa* into alginate-producing mucoid forms (Martin et al., 1993; Boucher et al., 1997).

There is no consensus on whether or not the CF ASL is substantially more acidic than those in normal subjects. We have been able to consistently obtain more acidic pH measurements in human ALI bronchial epithelial cell culture derived from ex-planted lungs of CF patients than those from non-CF subjects (Figure 4B). However, some studies reported that they were unable to measure significant pH differences in ASL of children w/wo CF (Schultz et al., 2017), and the pH values were highly dependent on the pH probe materials and locations of the measurement (nasal or tracheal) (McShane et al., 2003). Nonetheless, it was reported that without the innate proton pump ATP12A activity and airway acidification, CFTR-deficient mice are free from opportunistic *Staphylococcus aureus* respiratory infection (Shah et al., 2016), which makes airway acidification the deciding factor of murine opportunistic lung infections. In addition, eDNA originated from the bacterial biofilm and human immune cells also contribute to the acidification of CF microenvironments, which is evidenced by acidic pH gradients within *P. aeruginosa* biofilms (de los Rios et al., 2003; Hunter and Beveridge, 2005; Hidalgo et al., 2009) and acidified CF exhaled breath condensate (Tate et al., 2002; Ojoo et al., 2005), all of which are difficult to be discredited by the specific study using a direct ASL pH measurement in children, who are usually with less prominent CF phenotype and severity. Cowley et al. (2015) reported a pH range of 2.9 to 6.5 in pediatric CF sputum samples, which also disputes the notion that acidic pH is not a significant pathophysiological factor in CF. The CF microenvironment is much more complicated than what can be currently measured on human subjects. Our results are representative of the effects of an acidic environment on *P. aeruginosa* but do not address all abnormal CF conditions.

There are limitations to this study. We used the m63 medium for bacterial evolution experiments. M63 is a minimal medium that has been used for studying *P. aeruginosa* in CF (Wolfgang et al., 2004). We used D-glucose and 4 mM L-glutamine as the only nutrition sources in the m63 medium to maintain the pH consistency in simulating the CF-like acidic microenvironment. The synthetic cystic fibrosis sputum medium (SCFM) was developed to mimic the selective nutrient environment in the CF lungs (Palmer et al., 2007). However, we did not select SCFM for this study because it uses other amino acids, such as L-arginine, which causes an alkaline pH by releasing ammonia through deamination. While SCFM is a valuable tool in general CF-related studies, it remains an artificial medium and affects the acidic impact of cultivating *P. aeruginosa*. Therefore, we believe m63 is a more appropriate medium that consistently maintains acidic pH even after overnight bacterial culture. In our CFBE-ALI studies (Figure 4C), the PAO1 infected CFBE cells demonstrated a statistically significant decrease of CFU with ouabain treatment compared to normal saline control, but the averaged CFU number difference is relatively small. However, this is a proof of concept study. While the host-pathogen interaction model is more advanced than the conventional biofilm assay, this co-culture system still has limitations. For example, we could not incubate PAO1 on top of epithelial cells overnight to compare mature biofilm formation. Although the extended biofilm-forming period may show more substantial differences, the host cells would not be viable in the *in vitro* culture system after prolonged incubation with bacteria.

The choice between *P. aeruginosa* laboratory strains (PAO1 or PA14) throughout this study is justified for the following reasons. First, we used PAO1 instead of PA14 on CFBE-ALI studies because PA14 is highly virulent toward host cells by rapidly disrupting epithelial cell tight junction, as evidenced by our *Trans*-Epithelial Electrical Resistance (TEER) data (Supplementary Figure S5). PAO1 exhibits less acute virulence toward host cells compared to PA14 during the same time frame. Infecting epithelial cells with PAO1 better preserves the host cell integrity to facilitate the examination of biotic biofilm in the bacteria–host co-culture environment. Second, the ancestral PA14 did not increase its biofilm production under acidic pH without antibiotic pressure, but the PAO1 biofilm was significantly stimulated by acidic pH (Figure 3A and Supplementary Figure S2). It is commonly accepted that increased biofilm production contributes to antibiotic resistance. We determined that PA14 would be a better candidate for bacterial evolution in acidic pH to rule out the possibility that the rapidly accumulated antibiotic resistance is simply a result of increased biofilm formation.

We focused primarily on ceftazidime evolution and examined mutated PA14 at both population and clonal levels. We also sequenced the evolved PA14 populations from the ciprofloxacin or tobramycin treatments (Supplementary Figures S6A,B) at the end of the 15 days evolution and MIC monitoring (Figures 2D,E). In Supplementary Figure S6A, mutations on the *gyrA*, *nfxB*, and *morA* genes likely accounted for the primary resistance mechanism of PA14 toward ciprofloxacin in all 12 total populations (Wong et al., 2012; Ahmed et al., 2018; Farahi et al., 2018). Furthermore, we identified several specific mutations that existed only in an acidic environment (*pilU, fliF, orfN*, etc.). In Supplementary Figure S6B, tobramycin induced common mutations on *fusA* and *rplB* genes, which are proven mutations that contribute to PA14 tobramycin resistance (Feng et al., 2016; Scribner et al., 2020). Unlike the PA14 ceftazidime evolution study results where acidic pH-specific gene mutations contributed to antibiotic resistance, PA14 adapted to ciprofloxacin- and tobramycin-induced drug resistance by generating gene mutations in known mechanisms regardless of the pH conditions. These observations warrant further investigations on the mutation threshold of different classes of antibiotics. For instance, a single mutation on the PA14 *gyrA* gene could render ciprofloxacin useless while acidic pH promoted the resistance phenotypes even faster. The low cost of evolutionary trade-offs and easily mutated bacterial genome likely determine the performance of different antibiotics. Although the pH-specific genes are not individually investigated in this study, they are viable candidates that could guide future research that focuses on the mechanisms of pH-mediated bacterial drug resistance.

Of note, different culture media compositions could potentially impact antibiotic resistance and MIC results. For instance, Peng et al. (2015) provided evidence that higher exogenous alanine and/or glucose availability increases kanamycin susceptibility of some MDR pathogens. The underlying mechanism suggests that alanine or glucose promotes bacterial TCA cycle and proton motive force activity and, therefore, stimulates the bacterial intake of kanamycin. Such evidence suggests that bacterial culture media need to be carefully investigated before conducting drug-susceptibility tests. In this study, to appropriately measure bacterial growth curves with antibiotic treatment effects (Figure 1), which should include at least the first three phases of growth (lag phase, log phase, stationary phase, and death phase), we selected pH-adjusted tryptic soy broth (TSB) instead of m63 as the culture media. Although the growth curve data using m63 demonstrated antibiotic MIC differences in various pH similar to those using TSB media (Figure 1), the bacterial culture did not always reach the stationary phase for all tested *P. aeruginosa* within the same 18-h timeframe (data not shown). This culture media switch did not affect our conclusions but improved bacterial optical density measurements in a microplate reader. Interestingly, our results indicated that the acidic pH consistently stimulated PAO1 biofilm overproduction regardless of the use of different culture media were similarly shown in a separate study using Mueller Hinton broth (MHB), Luria-Bertani broth (LB), and TSB under different environmental conditions (Khan et al., 2020). It was also shown that acidic pH could enhance *in vitro* biofilm formation of *Streptococcus agalactiae* (D’Urzo et al., 2014) that correlated to a hypervirulent strain. It would be interesting in future studies to extensively evaluate the magnitude of physiologically relevant pH conditions that affect the resistance of different human pathogen species, in addition to the ESKAPE strains we examined in this study.

This study was designed to explore the mechanisms of initial *P. aeruginosa* colonization and how the CF-specific lung microenvironment might contribute to this adverse health outcome. *P. aeruginosa* colonization in respiratory tracts is a chronic process, and the bacteria likely adapt and evolve in the CF lungs for decades. Longitudinal studies of the CF *P. aeruginosa* genotypes have illustrated that *P. aeruginosa* undergoes extensive genomic DNA mutations to survive in the CF lungs (Smith et al., 2006). Although many mutated genes have been identified, there was no connection between any previously identified mutations to the acidic pH environment. Our WGS data provided direct evidence that correlates acidic pH-promoted bacterial drug resistance to short-term bacterial evolution. More studies are needed to target this aim and explore the role of acidic pH in long-term *P. aeruginosa* evolution/adaption to resolve the persistent biofilm formation and chronic colonization suffered by CF patients.

## Materials and Methods

### Bacterial Strains

Clinical *P. aeruginosa* strains were isolated from pediatric CF patients with chronic pulmonary infections at Seattle Children’s Research Institute except for AA43 and KK1 (Bragonzi et al., 2009), which were from a collection of the CF clinic at Medizinische Hochschule of Hannover, Germany. The lab strains used in this study were *P. aeruginosa* PAO1 (ATCC, BAA-47), UCBPP-PA14 (Rahme et al., 1995), *Staphylococcus aureus* ATCC#49775, *Klebsiella pneumoniae* ATCC#43816, and *Acinetobacter baumannii* ATCC#19606. All other ESKAPE species were collected from patients at the University of Pittsburgh Medical Center or Seattle Children’s Research Institute.

### Preparation of *Pseudomonas aeruginosa*

All bacteria were retrieved from −80°C glycerol stock and streaked on tryptic soy agar plates. Single colonies were picked and incubated in tryptic soy broth (TSB) overnight at 37°C in an orbital shaker. The overnight culture was diluted at 1:5 with fresh TSB and incubated for an additional 2 h for exponential growth. Bacteria were centrifuged at 2,000 × *g* for 5 min. The pellet was resuspended in 1 ml PBS. To ensure reproducible results, bacterial concentration was adjusted to approximately 10^9^ CFU/mL, optical density (OD_500 nm_) = 0.5 ± 0.01 in a spectrophotometer.

### Bacterial Growth Inhibition Assay

All GIA studies were performed in 10% TSB diluted in PBS. The 96-well plate was incubated at 37°C in a microplate reader for 18 h. Optical density (OD) at 570 nm was measured every hour with continuous double orbital shaking at 425 cycles per minute. The pH of the bacterial culture was adjusted to 6.0, 6.5, 7.0, and 7.5 using hydrochloride acid (HCl). The starting bacterial concentration in each treatment group was 10^6^ CFU/mL. All pH-adjusted media were filter-sterilized by a syringe filter unit (pore size: 0.22 μm, Millipore SLGP033RS) to ensure sterility.

### Biofilm Assay

The crystal violet biofilm staining method developed by O’Toole (2011) was used in this study with slight modifications. All biofilm studies were performed in pH adjusted m63 medium supplemented with 1 mM MgSO_4_, 25 μM FeCl_3_, 40 mM of D-glucose, and 4 mM of L-glutamine. The m63 was supplemented with 10% TSB to facilitate *Staphylococcus aureus* growth. The combination of D-glucose and L-glutamine allows the m63 to maintain its pH after *P. aeruginosa* overnight incubation. The testing bacterial concentrations were 5 × 10^7^ and 10^6^ CFU/ml for biofilm formation and antibiotic biofilm prevention, respectively. After 18 h of biofilm formation in a humidified 37°C incubator (with or without antibiotic treatment), the supernatant was carefully removed by pipetting, and biofilm attached to the plate was stained with 0.5% crystal violet (20% ethanol + 80% deionized water) solution for 15 min. The excessive dye was then rinsed off with water, and 95% ethanol was added to release the dye from the biofilm. OD values were acquired by a microplate reader at 620 nm wavelength.

### Bacterial Evolution in Antibiotics

Evolution studies were carried out using the m63 medium (same as biofilm assay). The bead transfer-based biofilm evolution model was described previously (Poltak and Cooper, 2011; Cooper, 2018; Jiang et al., 2019). Briefly, the PA14 ancestor was added to 5 mL of pH 6.5/7.5 m63 (media pH adjusted by HCl) with 1/2 MIC of antibiotic treatment at day 1. After 24 h, the PA14 biofilm (formed on a sterile acrylic bead) was transferred to the next tube of fresh m63 medium with a sterile bead inside. For planktonic culture, 50 uL of PA14 overnight culture was transferred to the next tube of fresh m63 medium. The dosage of antibiotics was doubled at the time of each transfer. The groups that survived antibiotic treatment were transferred to the next tube. The groups that could not tolerate the elevated antibiotic concentration were incubated again at the prior concentration without doubling antibiotic concentration.

### Bacterial Whole-Genome Sequencing

Bacterial genomic DNA samples were extracted using the Qiagen DNeasy PowerBiofilm Kit. Biofilm attached to beads was dissociated by sonication in sterile PBS before DNA extraction. Planktonic cultures were centrifuged and pelleted before DNA extraction. Library preparation (Baym et al., 2015; Turner et al., 2018) and WGS were performed by the University of Pittsburgh Microbial Genome Sequencing Center using Illumina NextSeq500.

### Bioinformatics

Raw sequencing reads were quality filtered and trimmed by Trimmomatic (Bolger et al., 2014) using the following parameters: NexteraPE-PE.fa:2:30:10 LEADING:20 TRAILING:20 SLIDINGWINDOW:4:20 MINLEN:70. Genetic variants were predicted by *breseq* v0.33.0 (Deatherage and Barrick, 2014). The *Pseudomonas aeruginosa* UCBPP-PA14 reference genome was downloaded from NCBI. The PA14 ancestor clone was sequenced to eliminate background mutations. The sequencing depths of all evolved PA14 populations were at least 125×. Mutation percentage higher than 20% are shown in the figures.

### Primary Airway Epithelial Cell Cultures

Differentiated primary human bronchial epithelial cells were derived from lungs removed at the time of lung transplantation at the Center for Organ Recovery and Education (Pittsburgh, PA, United States). Cells were prepared using previously described methods (Liu et al., 2013a, b) approved by the University of Pittsburgh Institutional Review Board. Donor primary human CF and non-CF bronchial epithelial cells were first isolated from donor tissues and propagate under submerged cell culture. Upon confluence, epithelial cells were disassociated and seeded onto a transwell cell culture plate at approximately 2 × 10^5^ cells/well (Corning, NY, United States). Epithelial cells were maintained in Bronchial Epithelial Cell Growth Medium (Lonza, Basel, Switzerland). The cell culture was changed to ALI by removing the apical medium 3 days after initial cell seeding and maintained for 3 weeks for cell differentiation.

### Treatment and Host Defense Activity of Primary Airway Epithelial Cell Cultures

The 4 mM ouabain stock solution was prepared and dissolved in DMSO. Ouabain was diluted to 20 μM in normal saline and 20 μL of the diluted ouabain or DMSO (vehicle control) was added to the primary cell cultures apically and incubated for 2 h in 37°C supplied with 5% CO_2_. Primary cultured epithelial cells were washed apically with 100 μL of PBS 24 h before the experiment. A total of 10 μL of the apical fluid was immediately added onto pH test strips. After pH reading, all remaining apical fluid was removed, and another 20 μL of 20 μM ouabain or DMSO was added with 50 μL of PAO1 suspended in normal saline (10^7^ CFU/insert). Cells were then incubated for additional 5 h for biofilm to form. All apical supernatant was collected for determining the CFU of unattached planktonic bacteria. Biotic biofilm assay was used by counting CFU of bacterial biofilm formed on the epithelial cells. ALI membrane was removed from the filter and sonicated in 2 mL of PBS for 30 s at 80% amplitude by the DPS-20 dual processing system (130 W; PRO Scientific) to disassociate congregated PAO1 biofilm. After sonication, both planktonic and biofilm samples of PAO1 were plated on tryptic soy agar plates for total CFU counting.

### Gene Expression

A total of 5 × 10^7^ CFU/mL PAO1 was cultured in pH-adjusted (pH = 6.0, 6.5, 7.0 and 7.5) DMEM for 3 h in 100 × 15 mm round-petri dishes at 37°C. For biofilm RNA samples, supernatant in all petri dishes was discarded and a thin layer of PAO1 biofilm was scraped off using a sterile cell scraper. The same amount of PAO1 was incubated separately in 5 mL of pH 7.5 DMEM at the same time and served as a planktonic control. Total biofilm and planktonic RNA were extracted as previously published (Cury and Koo, 2007; Lin et al., 2018; Casciaro et al., 2019). The cDNA was synthesized by High-Capacity cDNA Reverse Transcription Kit (Applied Biosystems). Gene expression results were obtained using the Fast SYBR Green Master Mix (Applied Biosystems) and the 7900HT Fast Real-Time PCR System (Applied Biosystems). ΔΔ*C*_t_ values were calculated and analyzed using a method previously published (Lin and Di, 2020; Lin et al., 2021). The list of primers is listed in Supplementary Table S2.

### Bacterial Motility Assay

Bacterial swarming and twitching motility assays were performed according to the protocols previously published (Ha et al., 2014; Turnbull and Whitchurch, 2014). M8 medium (Na_2_HPO_4_⋅7H_2_O, supplemented with 0.5% casamino acids, 0.2% D-glucose, 1 mM MgSO_4_) was used for agar preparation (0.5% agar for swarming and 1.5% agar for twitching). Agar pH was adjusted to 7.5 and 6.5 by concentrated HCl. The overnight culture of PAO1 was diluted to 10^6^ CFU/mL. For swarming motility, 2.5 μL of diluted PAO1 was dropped to the center of the agar. For twitching motility, a thin 10 μL pipette tip was first dipped into the diluted PAO1, then punctured through the center of the agar. Plates were incubated at 37°C for 24 h before measurement.

### Measurement of *Trans*-Epithelial Electrical Resistance on Human Bronchial Epithelial Cell Cultures

The human WT-CFTR bronchial epithelial cells were cultured in Minimum Essential Medium supplemented with 10% fetal bovine serum and 0.5 μg/mL of puromycin in a 37°C incubator supplemented with 5% CO_2_. A total of 2 × 10^5^ cells were seeded on each ALI insert. The apical medium was removed after 3 days to convert to an air-liquid interface. Cells were used for TEER measurements after 7 days. The overnight cultures of PAO1 and PA14 were diluted in PBS to a final concentration of 10^8^ CFU/mL. A total of 100 μL diluted bacteria were added to the apical side of the ALI. TEER was measured using the Millicell^®^ ERS-2 Voltohmmeter (Millipore Sigma, MERS00002).

### Statistical Analysis

Error bars represent mean ± standard error of the mean (SEM). One-way analysis of variance (ANOVA) and Tukey’s multiple comparisons tests were used to assess the overall change of MIC in the bacterial evolution model. One-way ANOVA and Dunnett’s multiple comparisons test were used to assess the change of bacterial gene expressions among various pH conditions. Two-way ANOVA and Dunnett’s multiple comparisons test were used to assess the effects of pH and salt on bacterial biofilm formation. Student’s *t*-test was used to assess statistical significance between two subjects, such as biofilm formation and antibiotic biofilm prevention in different pH, human ALI ASL pH, and CFU with or without ouabain treatment. ^∗^*p* < 0.05; ^∗∗^*p* < 0.01; ^∗∗∗^*p* < 0.001; ^∗∗∗∗^*p* < 0.0001; otherwise not significant (NS).

## Data Availability Statement

The sequencing data have been deposited with links to BioProject accession number PRJNA685187 in the NCBI BioProject database (https://www.ncbi.nlm.nih.gov/bioproject/PRJNA685187).

## Ethics Statement

The studies using primary non-CF and CF HBE cells obtained from explanted lungs of patients with written informed consent under a protocol reviewed and approved by the Institutional Review Board at the University of Pittsburgh.

## Author Contributions

YD contributed to the conceptualization, funding acquisition, and supervision. QL, JP, and YD contributed to the methodology. QL and YD contributed to the investigation. QL contributed to the writing – original draft. JP and YD contributed to the writing – review and editing and resources. All authors contributed to the article and approved the submitted version.

## Conflict of Interest

The authors declare that the research was conducted in the absence of any commercial or financial relationships that could be construed as a potential conflict of interest.

## Publisher’s Note

All claims expressed in this article are solely those of the authors and do not necessarily represent those of their affiliated organizations, or those of the publisher, the editors and the reviewers. Any product that may be evaluated in this article, or claim that may be made by its manufacturer, is not guaranteed or endorsed by the publisher.
