# Supplementary material for: Acidic Microenvironment Determines Antibiotic Susceptibility and Biofilm Formation of Pseudomonas aeruginosa
Source: Front Microbiol. 2021 Nov 19;12:747834. doi: 10.3389/fmicb.2021.747834 (PMC8640179; doi:10.3389/fmicb.2021.747834)
Supplement: Supplementary file 1 [file Data_Sheet_1.pdf]

Supplementary data

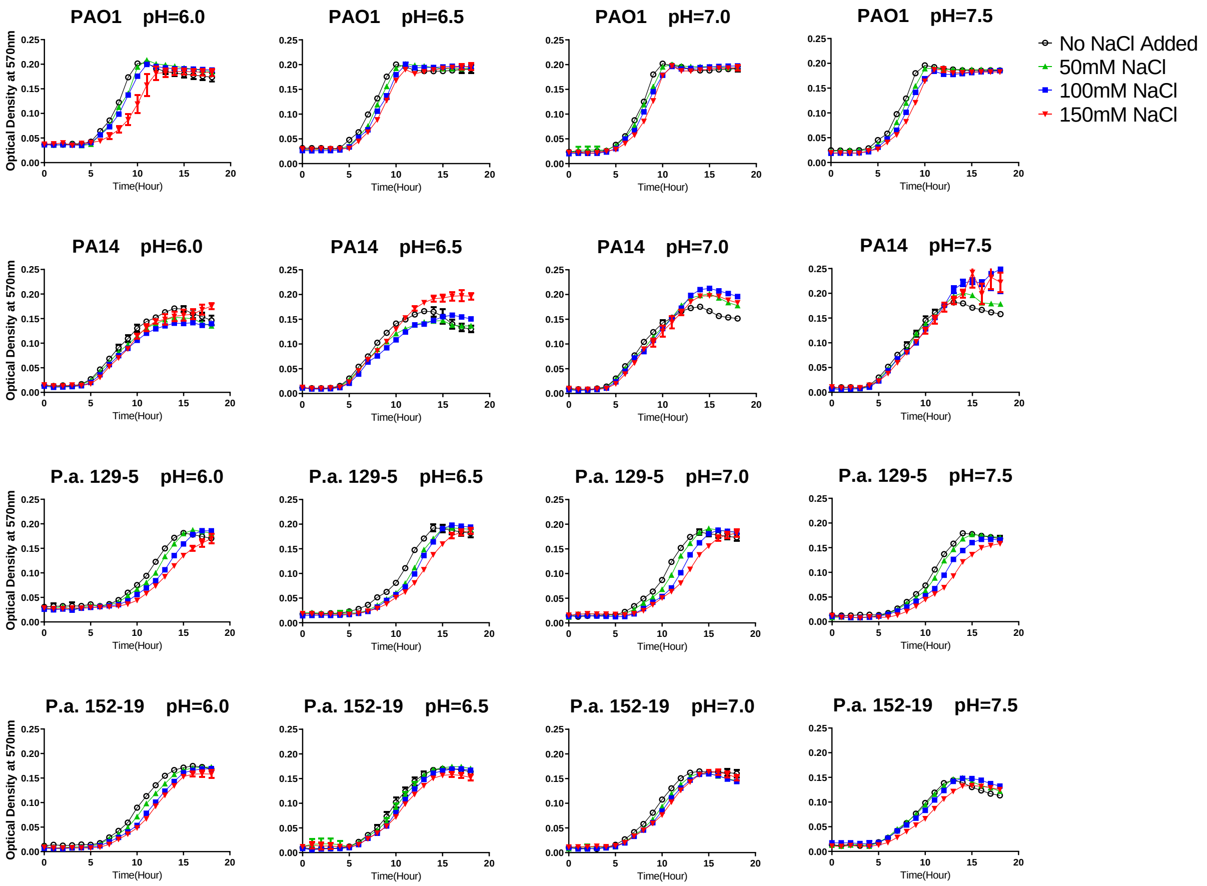

Fig. S1. Adjusted salt and pH conditions do not significantly affect the proliferation rate of planktonic *P. aeruginosa*. PAO1, PA14, *P.a.*129-5 and *P.a.*152-19 were inoculated in 10% TSB (n=3). The salt concentration of the culture media was adjusted by the addition of 50, 100, and 150mM NaCl. No NaCl group (pH=7.5) was used as a control. Optical density at 570nm was measured every hour for 18 hours at 37°C in a microplate reader.

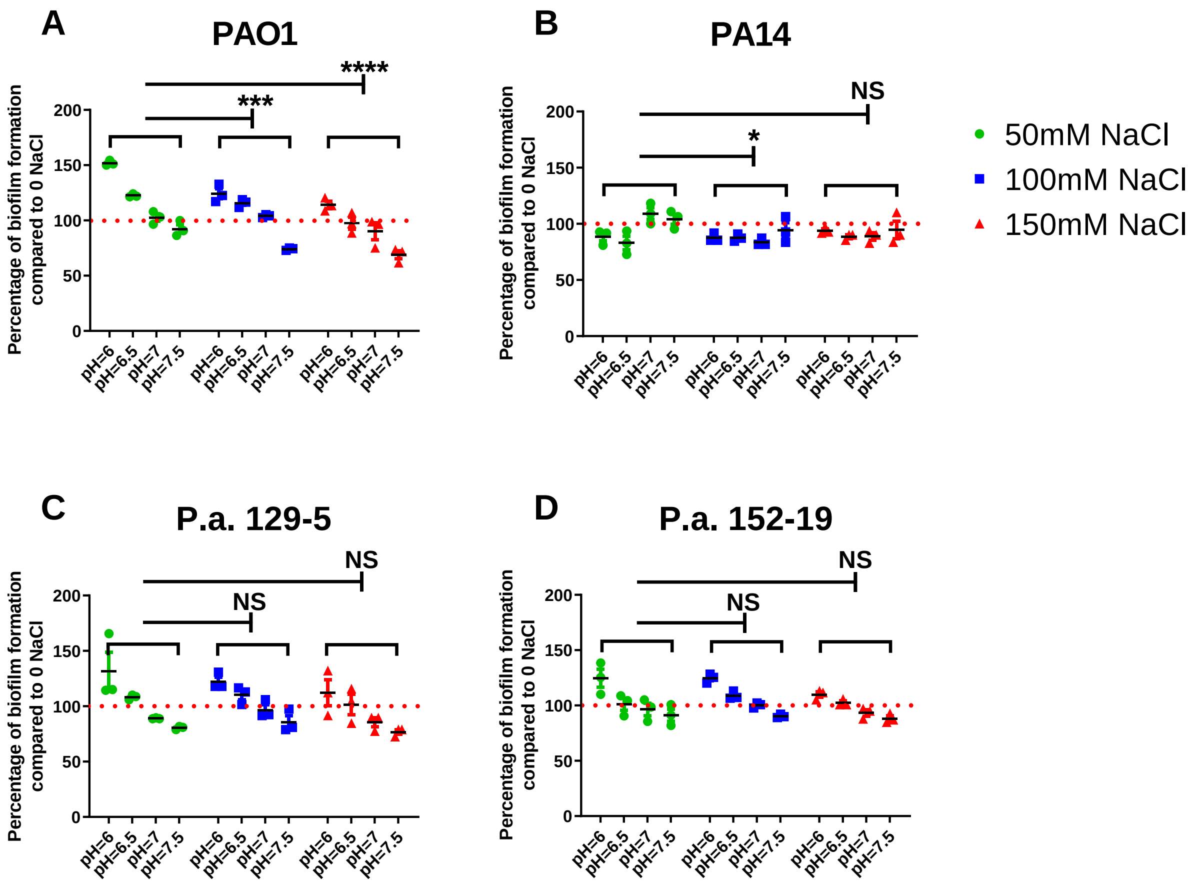

Fig. S2. Elevated NaCl concentrations have minimal effect on *P. aeruginosa* biofilm formation but acidic pH promotes *P. aeruginosa* biofilm formation. (**A** to **D**) Effect of NaCl (50, 100 and 150mM) and pH (6.0, 6.5, 7.0 and 7.5) on *P. aeruginosa* biofilm formation compared to positive controls (n=3). Bacteria were incubated for 3 hours at 37°C in 96-well microplates. The crystal violet staining method was used to quantify the biofilm/biomass attachment. The red dotted lines denote the (+) control of biofilm formation at pH 7.5 without additional NaCl. Data are mean ± SEM. Two-way ANOVA was used for statistical analysis. $^{**}p<0.01$; $^{***}p<0.001$; $^{****}p<0.0001$; NS: not significant.

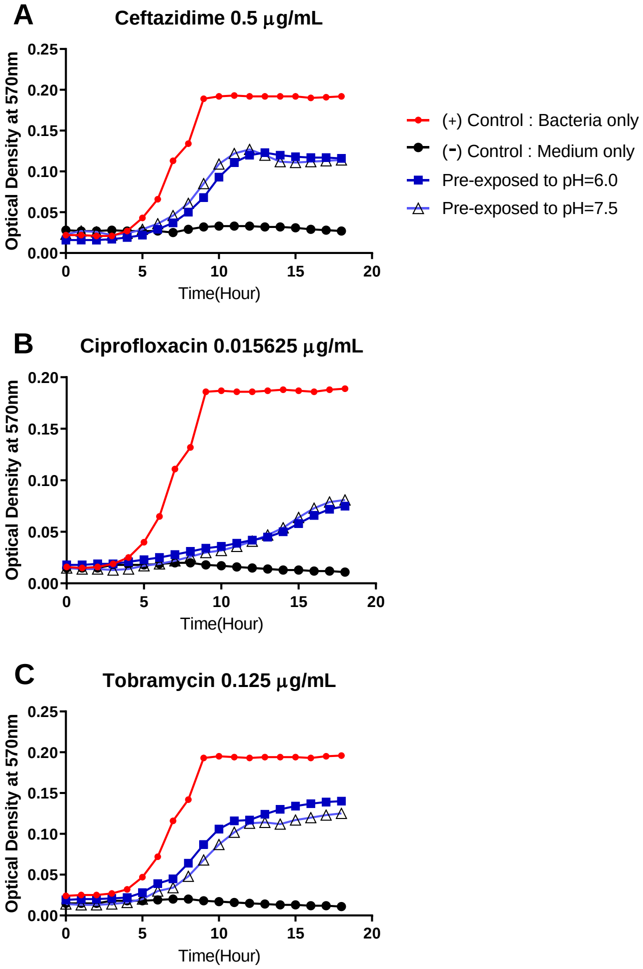

Fig. S3. Antibiotics restore their antimicrobial activity in physiological pH conditions after prior exposure to an acidic environment. Antibiotics (ceftazidime, ciprofloxacin, and tobramycin) were pre-exposed to pH = 6.0 or 7.5 in 10% TSB for 5 hours, the pH for all exposed antibiotics was then re-adjusted back to 7.5 before the antibiotics were added into planktonic *P. aeruginosa* (PAO1) culture for GIA at pH 7.5. The applied treatment concentration for each antibiotic was selected using less than their respective MIC to demonstrate the partial inhibition of bacterial growth. Optical density at 570nm was measured every hour for 18 hours at 37°C in a microplate reader. Results are mean ± SD from two independent experiments with bacteria grew in duplicates for each condition.

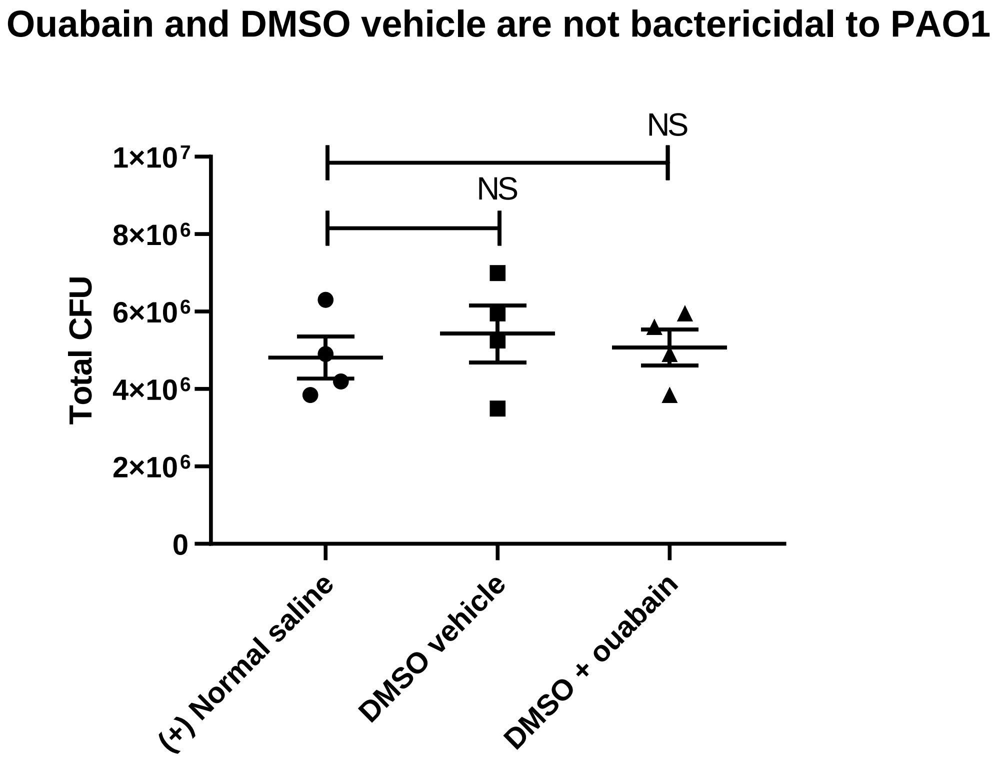

Fig. S4. Ouabain treatment was not bactericidal to PAO1. A total volume of 50µL of 2 x 10^8 CFU/mL PAO1 and 20µL of 20µM Ouabain or DMSO (solvent control) were incubated in normal saline for 5 hours at 37°C (n=4). Data are mean ± SEM from two repeated experiments. Student's *t*-tests were used for statistical analysis. NS: not significant.

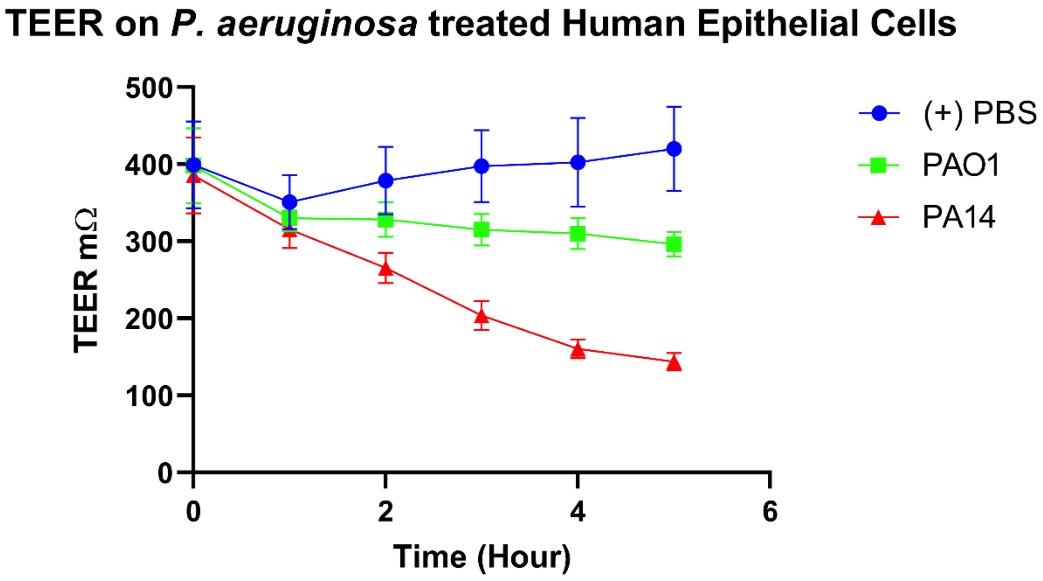

Fig. S5. Comparison of acute bacterial virulence on CFBE cells. Polarized CFWT airway epithelial cells on the air-liquid interface were treated with PBS, PAO1, or PA14. Trans-epithelial electrical resistance (TEER) was measured every hour for 5 hours. Positive (+) control is PBS only (on apical sides). Data were collected from two independent experiments (n=4).

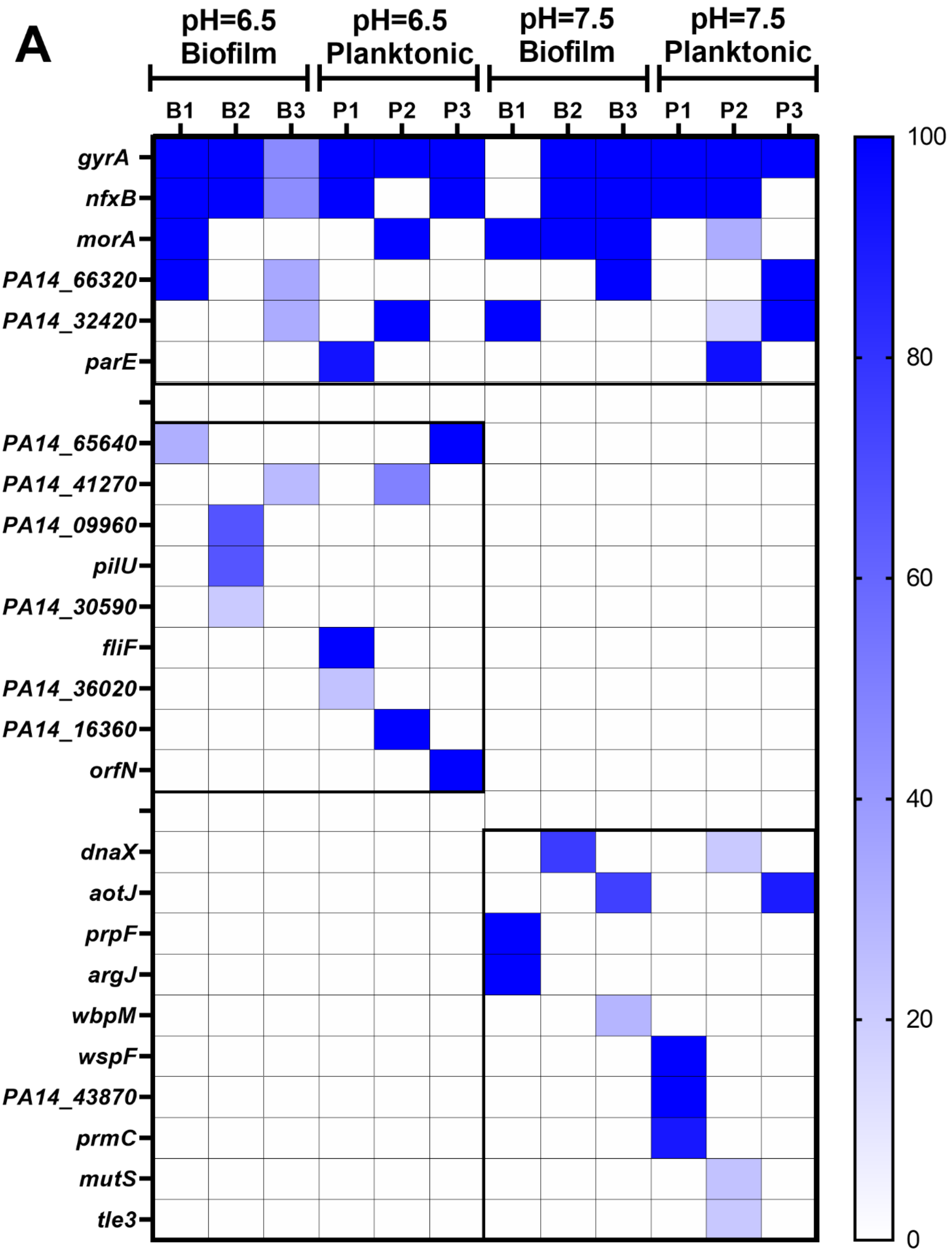

A
pH=6.5
Biofilm
pH=6.5
Planktonic
pH=7.5
Biofilm
pH=7.5
Planktonic
B1
B2
B3
P1
P2
P3
B1
B2
B3
P1
P2
P3
gyrA
nfxB
morA
PA14_66320
PA14_32420
parE
PA14_65640
PA14_41270
PA14_09960
pilU
PA14_30590
fliF
PA14_36020
PA14_16360
orfN
dnaX
aotJ
prpF
argJ
wbpM
wspF
PA14_43870
prmC
mutS
tle3
100
80
60
40
20
0

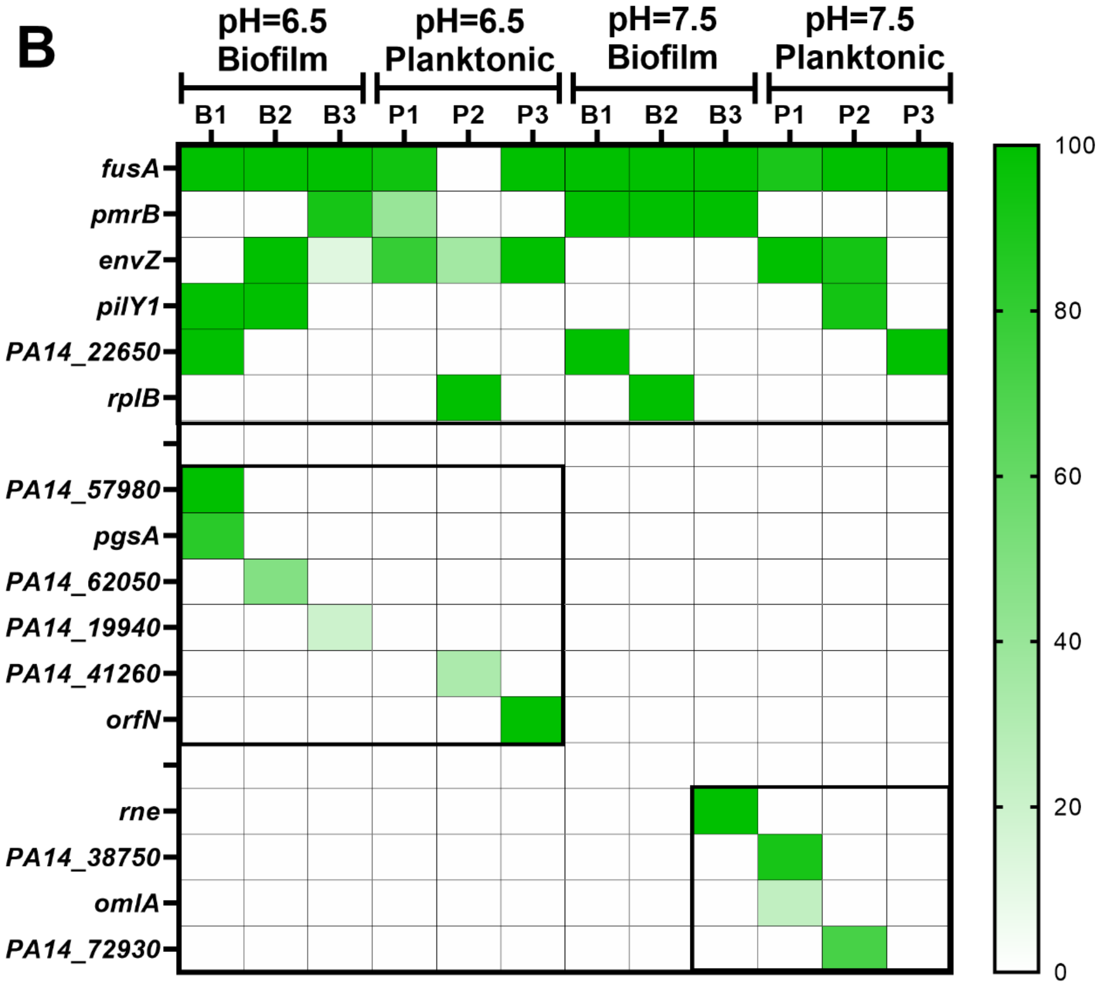

Fig. S6. PA14 population mutations were identified after 15 days of evolution with ciprofloxacin or tobramycin. (**A**) Mutations detected from the ciprofloxacin treated PA14 populations; (**B**) Mutations detected from the tobramycin treated PA14 populations. Each bacterial lifestyle/pH was evolved in triplicate populations. Biofilm (B) /planktonic (P) populations were labeled as B1, B2, B3, and P1, P2, P3, respectively. Color scale bars represent mutation frequency, which ranges from 0 to 100%.

Supplementary Table 1. Effects of acidic pH on *P. aeruginosa* antibiotic tolerance in MIC changes. MIC data were acquired using the GIA assay. Results are averaged MICs from two independent GIA experiments.

Ceftazidime MIC (μg/mL)				
NO.	Strain	pH 6.5 average MIC	pH 7.5 average MIC	MIC fold increase from pH 6.5 to 7.5
1	PAO1	1.875	0.9375	2
2	PA14	2.5	1.25	2
3	116-37	2.5	0.9375	3
4	71-75	0.625	0.15625	4
5	KK1	2.5	1.25	2
6	AA43	5	2.5	2
7	40-21	2.5	1.25	2
8	151-40	5	2.5	2
9	129-5	5	1.25	4
10	109-10	2.5	1.25	2
11	480-1	0.625	0.15625	4
12	82-9	2.5	1.25	2
13	152-19	5	2.5	2
14	80-37	2.5	1.25	2

Ciprofloxacin MIC (μg/mL)				
NO.	Strain	pH 6.5 average MIC	pH 7.5 average MIC	MIC fold increase from pH 6.5 to 7.5
1	PAO1	0.0390625	0.0390625	1
2	PA14	0.07813	0.0390625	2
3	116-37	0.07813	0.0390625	2
4	71-75	0.15625	0.1171875	1
5	KK1	0.03906	0.0390625	1
6	AA43	0.15625	0.078125	2
7	40-21	0.07813	0.0390625	2
8	151-40	0.9375	0.625	2
9	129-5	0.07813	0.0390625	2
10	109-10	0.15625	0.05859375	3
11	480-1	0.01464845	0.0146485	1
12	82-9	0.07813	0.0390625	2
13	152-19	0.07813	0.0390625	2
14	80-37	0.0390625	0.0390625	1

Tobramycin MIC (μg/mL)				
NO.	Strain	pH 6.5 average MIC	pH 7.5 average MIC	MIC fold increase from pH 6.5 to 7.5
1	PAO1	2.5	0.9375	3
2	PA14	3.75	1.25	3
3	116-37	1.25	0.3125	4
4	71-75	2.5	0.625	4
5	KK1	5	1.25	4
6	AA43	10	1.875	5
7	40-21	1.25	0.3125	4
8	151-40	2.5	1.25	2
9	129-5	0.625	0.625	1
10	109-10	10	2.5	4
11	480-1	0.9375	0.3125	3
12	82-9	5	0.625	8
13	152-19	1.25	0.625	2
14	80-37	5	0.9375	5

Supplementary Table 2. List of primers used for quantitative real-time PCR.

Gene	Primer sequence	
tolA	Forward	5’ - GCG TAA TGG AAT GAG CGT AGA A -3’
	Reverse	5’ - CGA ACT GTC GAA AGG CTT GT - 3’
ndvB	Forward	5’ - TGT GGA TCG CCT ACG ACT A - 3’
	Reverse	5’ - CGG TGA ACA GCA CGA TGA - 3’
mucB	Forward	5’-TTT GCT TGG CAG CCT GAT-3’
	Reverse	5’-GGT GCC TTG GAA ACT GTT CT-3’
exoS	Forward	5’-GAC AGG CTG AAC AGG TAG TG-3’
	Reverse	5’-TTC AGG GAG GTG GAG AGA TAG-3’
exoT	Forward	5’-GCT GAA CAG GTC GTG AAG A-3’
	Reverse	5’-CCG GGA GGT GGA GAG ATA G-3’
fimT	Forward	5’-CGC TTG CAA AGA AGG AAA GG-3’
	Reverse	5’-CCT TCC GCA GAG CAG AAA-3’
fimX	Forward	5’-CCT GGC CTA TAT CCA TCT CAA C-3’
	Reverse	5’-ACT GTT CAC GCA TCA GTC C-3’
rhlA	Forward	5’-CGA GAC CGT CGG CAA ATA C-3’
	Reverse	5’-GCA CCT GGT CGA TGT GAA A-3’
rhlB	Forward	5’-CTC ACG AGA AGT ACG GGA TTC-3’
	Reverse	5’-CTC GGG CAC GTT GAA CT-3’
rplU *	Forward	5’-CGC AGT GAT TGT TAC CGG TG-3’
	Reverse	5’-AGG CCT GAA TGC CGG TGA TC-3’

*The constitutively expressed *rplU* gene served as a reference gene.
